# Prevention of road traffic collisions and associated neurotrauma in Colombia: An exploratory qualitative study

**DOI:** 10.1371/journal.pone.0249004

**Published:** 2021-03-25

**Authors:** Santhani M. Selveindran, Gurusinghe D. N. Samarutilake, David Santiago Vera, Carol Brayne, Christine Hill, Angelos Kolias, Alexis J. Joannides, Peter J. A. Hutchinson, Andres M. Rubiano

**Affiliations:** 1 Department of Clinical Neurosciences, Addenbrooke’s Hospital, Cambridge, United Kingdom; 2 NIHR Global Health Research Group on Neurotrauma, University of Cambridge, Cambridge, United Kingdom; 3 Directorate of Healthcare Quality and Safety, Ministry of Health, Colombo, Sri Lanka; 4 Institute of Public Health, University of Cambridge, Cambridge, United Kingdom; 5 Meditech Foundation, Cali, Colombia; 6 Neuroscience Institute, Universidad El Bosque, Bogotá, Colombia; Universitat de Valencia, SPAIN

## Abstract

**Introduction:**

Neurotrauma is an important but preventable cause of death and disability worldwide, with the majority being associated with road traffic collisions (RTCs). The greatest burden is seen in low -and middle- income countries (LMICs) where variations in the environment, infrastructure, population and habits can challenge the success of conventional preventative approaches. It is therefore necessary to understand local perspectives to allow for the development and implementation of context-specific strategies which are effective and sustainable.

**Methods:**

This study took place in Colombia where qualitative data collection was carried out with ten key informants between October and November 2019. Semi-structured interviews were conducted and explored perceptions on RTCs and neurotrauma, preventative strategies and interventions, and the role of research in prevention. Interview transcripts were analysed by thematic analysis using a framework approach.

**Results:**

Participants’ confirmed that RTCs are a significant problem in Colombia with neurotrauma as an important outcome. Human and organisational factors were identified as key causes of the high rates of RTCs. Participants described the current local preventative strategies, but were quick to discuss limitations and challenges to their success. Key barriers reported were poor attitudes and knowledge, particularly in the community. Suggestions were provided on ways to improve prevention through better education and awareness, stricter enforcement and new policies on prevention, proper budgeting and resource allocation, as well as through collaboration and changes in attitudes and leadership. Participants identified four key research areas they felt would influence prevention of RTCs and associated neurotrauma: causes of RTCs; consequences and impact of RTCs; public involvement in research; improving prevention.

**Conclusion:**

RTCs are a major problem in Colombia despite the current preventative strategies and interventions. Findings from this study have a potential to influence policy, practice and research by illustrating different solutions to the challenges surrounding prevention and by highlighting areas for further research.

## Introduction

Neurotrauma is an important cause of death and disability worldwide [1]. Recent studies estimate that the annual global incidence of neurotrauma ranges from 500 to 800 per 100,000 per year [2, 3]. The consequences of neurotrauma are widespread, due to the negative impact on a country’s economy, healthcare system, society and demography [4–6]. This is especially so for low -and middle- income countries (LMICs) which experience the greatest burden of this ‘silent epidemic’ [2, 6, 7].

Road traffic collisions (RTCs) have been identified as the largest cause of neurotrauma [7]. Rapid urbanisation and motorisation have made this a major problem in LMICs, where 90 percent of road traffic injuries and deaths occur [7, 8].

RTCs and thus, neurotrauma are predictable, preventable and could be mitigated if the appropriate strategies are put in place at societal, community, household and individual levels [6, 9]. Preventative programmes should be developed with strong scientific evidence to maximise effectiveness [10]. This is particularly important for LMICs where a ‘one size fits all’ approach would not always be feasible due to observed variations in the environment, infrastructure, population and habits [11, 12]. An in-depth understanding of local circumstances and needs is imperative for guiding policy and practice as it allows for appropriate resource allocation, and facilitates the development and implementation of context-specific, yet effective, interventions [13–16].

Engaging with people and groups affected by the issues can help glean such information. One method that has been utilised for this purpose is key informant interviews [13, 17]. Key informants are considered as central figures who may serve a variety of professional or personal functions within a community [17]. These individuals are expected to have good understanding of local conditions and are reasonably knowledgeable about a particular subject [17, 18]. Hence, key informant interviews provide a broad, informative overview of the problem or issue that needs to be addressed, as well as identify potential solutions [17, 19].

The current evidence-base shows that such engagement in the area of prevention of RTCs and neurotrauma is lacking in LMICs [7, 11, 12]. To address this gap, the Global Prevention of NeuroTrauma-Road Traffic Collisions (GPONT-RTC) research project was developed. This multi-centre project involves several research aims in LMICs, one of which is to illustrate the current context within which RTCs and neurotrauma from RTCs take place, and describe factors that need to be prioritised for formulating and implementing successful prevention programmes.

Colombia is one of the countries participating in this research project. As with other LMICs, RTCs are an important public health problem in this country [20]. Although policies and programmes have been put in place over the years to reduce the high rates of injury and death, there is still a lack of clear information on the true magnitude of the problem and on the usefulness of the implemented approaches [20, 21]. Understanding and exploring these issues from a local perspective, through key informant interviews, not only facilitates prioritisation of efforts and resources, but is also critical for sustainability [12–14, 21]. As a study that is situated within a larger body of work, the aim of this paper was to explore prevention of RTCs and associated neurotrauma in Colombia from the perspectives of key informants.

## Methods and materials

### Setting and design

This present study took place in Colombia, a country situated in the north of South America with a population of 49.6 million [22]. According to the World Bank economic classification, Colombia is an LMIC in the Upper middle income group [23].

This study utilised a descriptive qualitative approach and followed the consolidated criteria for reporting qualitative research (COREQ) checklist [24]. The completed checklist is available in the S1 File. The descriptive approach was selected as this study sought to learn about people’s perspectives or experiences of phenomenon, rather than theory-generation [25, 26]. This approach enables the researcher to stay close to participants’ viewpoints on a particular issue or phenomenon and provide a rich description of the experience in easily understood language [27].

### Sample and recruitment

Given our aim and study approach, we carried out purposive sampling of key informants who have the requisite knowledge and experience of the phenomenon under investigation [27].

Selection criteria for the key informants (KIs) were based on their involvement and previous experience in neurotrauma and/or RTCs prevention. Participants were selected from the following categories: 1) Commissioning Stakeholders; 2) Service providers; 3) Community/local patient/advocacy group representatives.

Potential participants were identified from a list compiled by the MEDITECH Foundation in Colombia, an organisation two of the authors (DSV and AMR) had affiliations with. MEDITECH Foundation (Fundación MEDITECH) is a non-governmental collaborative organisation which is involved in research and education relating to emergency and disaster care (see https://www.meditechfoundationglobal.org).

MEDITECH Foundation (Fundación MEDITECH) is a non-governmental collaborative organisation which is involved in research and education relating to emergency and disaster care (see https://www.meditechfoundationglobal.org).

Potential participants were invited to be involved in this study by the interviewer (DSV) via telephone, and were briefed on the study and consenting requirements.

Of the ten participants in the final sample, two were commissioning stakeholders, 6 were service providers and two were community representatives, where one also represented a non-governmental organisation (NGO). All the selected participants had more than 5 years’ experience in RTCs-related and/or neurotrauma prevention.

### Data collection

Semi-structured interviews were carried out in Spanish either face-to-face or via telephone, using a standardised interview guide. The interview guide, originally developed in English as part of the GPONT-RTC multi-centre study, was translated into Spanish by the local research team. Back-translation of the translated interview guide was carried out by an independent party to ensure linguistic validity and reduce bias. The guide was pre-tested on two participants and refined.

The main guiding questions developed to address the research objective are given in Table 1. Additional question options were also added to extract other relevant information. The interviewer (DSV), is a medical doctor with training and experience in qualitative methods, who now works as a full-time researcher at MEDITECH Foundation, and is a collaborator on the GPONT-RTC research project. The interviewer did not have any contact with any of the research participants prior to the recruitment process. The interviews were conducted in the form of a discussion to facilitate data flow while establishing good rapport with the participants.

**Table 1:** Main guiding questions.

1. What are the perceptions of key informants on RTCs in their city/country and its role in neurotrauma causation?
2. What is the understanding of key informants on neurotrauma prevention, and their perceptions on neurotrauma and RTCs prevention in their city/country?
3. What is the understanding of key informants on current local research in neurotrauma and RTCs prevention, and what are the research priorities in this area?

Interviews, which took place between October and November 2019, were audio-recorded and lasted around 10 to 30 minutes, with additional notes written up after their completion. Only the interviewer and participant were present during the interviews, that were carried out while the participants were at work. This was a single interview design and no repeat interviews were carried out.

### Data management and analysis

Audio-recordings were translated into English and transcribed by an authorised professional service. The translated transcripts were checked by the local team to ensure accuracy. Edits were made where necessary.

The transcripts were analysed manually by thematic analysis using a framework approach [28]. As an analytical approach that sits within the broad family of thematic analysis and is not aligned to any particular philosophical or theoretical approach, this method is suitable for qualitative descriptive studies where it allows for straightforward, transparent results that remain close to the ‘surface of the data’, and is shaped by existing ideas without the need to produce a new theory [25, 27–30]. This approach can also be easily applied without the need for qualitative data analysis software [29].

The steps in this method encompass familiarisation with the interview data, coding, developing a working analytical framework, indexing transcripts using the analytic framework, charting data into the framework matrix, and interpreting the data [28].

After familiarisation with the transcripts, deductive and inductive codes were generated using the first five transcripts. These codes were used to develop an analytic framework, consisting of initial themes and sub-themes, which was refined and finalised after coding of the last transcript, and no new codes were identified. The analytical framework was applied to each transcript where relevant portions which corresponded to a particular theme were identified. This allowed for further refinement of the themes and sub-themes in order to accurately reflect the data, whilst aligning to research objectives. These sections of data were summarised and incorporated into thematic charts. This exercise facilitated the process of data interpretation where any connections or relationships between categories were mapped, and the final themes and sub-themes were generated.

To ensure validity, peer checking was carried out for each step of the analysis, where the data was analysed independently by two researchers (SMS and GDNS). Analytical discrepancies were resolved through a consultative approach.

### Ethics

Ethical approval for this study was obtained from the Research Ethics Committee of the MEDITECH Foundation and the University of Cambridge School of Humanities and Social Sciences (Case no. 19/204). Informed consent was obtained from all study participants either verbally or in writing, depending on how they were interviewed. The consent also covered permission for audio-recording of the interviews. Prior to each interview, the interviewer explained the study to the participants using the details found in the information sheet. To maintain participant confidentiality, audio-recordings and transcripts were stored securely, and were only accessible to the interviewer and the researchers involved in data analysis. Also, any identifiable information was anonymised during the reporting of research findings.

## Results

Interviews were conducted with ten KIs (7 female, 3 male). KIs resided in the cities of Cali, Yopal and Ibague, and the towns of Andalucia and Cajamarca.

From the analyses, two overarching themes were identified as shown in Table 2. As a result of the translation, the terms ‘traffic accidents’ or ‘accidents’ are found in the quotes, instead of RTCs or ‘collisions’, and these will be used interchangeably.

**Table 2:** Themes and sub-themes from key informant interviews.

Theme	Sub-theme
1. Significance of RTCs in Colombia	1.1.Distribution of RTCs
	1.2. Determinants of RTCs
	1.3. Impact of RTCs
2. Preventing neurotrauma and RTCs in Colombia	2.1.Perceptions of neurotrauma prevention
	2.2. Current preventative strategies and interventions
	2.3. Barriers to prevention
	2.4. Improving prevention
	2.5. Role of research

### Significance of RTCs in Colombia

#### Distribution of RTCs

All participants indicated that there are many RTCs taking place in their city and country as a whole. RTCs tend to occur frequently, usually on a daily basis, and although these are more common in towns, there are also occurrences in villages. These RTCs tended to involve motorcycles, with many of the riders being young people.

*“Traffic accidents are of great importance in Colombia*. *There are too many on a regular basis…*. *There are car and motorbike accidents*. *Here where we are*, *in the centre of town*, *it seems to be on a daily basis*, *but I believe it’s very frequent everywhere*.*”* (KI7, Community representative)

“… *there are many accidents frequently*, *especially motorbikes…*.*we have a very high number of motorbike accidents*. *There are many motorcycle accidents in this region of the country*…*Most accidents are motorbikes against cars*..*”* (KI9, Commissioning Stakeholder)

*“There are many accidents in town and most are motorcycles and the drivers tend to be young*.*”* (KI 1, Service Provider)

“*Here in [*..*] we’ve had many traffic accidents…Many youngsters driving motorbikes…there are so many accidents involving young people*.”(KI10, Commissioning Stakeholder)

#### Determinants of RTCs

Participants identified various reasons for RTCs and related injuries in Colombia. Causes resulting from Individual, Inter-personal and Community, and Government and Organisational factors emerged from the interviews.

*Individual factors*. There was consensus among participants that RTCs occurred due to the behaviour of road users which they characterised as careless, reckless, irresponsible and inconsiderate. Driving under the influence of alcohol and/or substances, and speeding were identified as being the main risk factors leading to RTCs.

“..*most accidents are caused by drinking alcohol*, *overtaking and not adhering to the law*.*”* (KI 2, Service Provider)

This was often seen in conjunction with the absence or improper use of personal protective equipment.

*“*..*there are so many accidents involving those who drink too much because that’s actually one of the main causes of accidents in this county*. *People are drunk and that causes traffic accidents*. *They don’t wear helmets and then suffer when they have an accident*.*”* (KI 10, Commissioning Stakeholder)

.*”we see a motorbike and the person will not be wearing a helmet or if they are*, *the helmet is not fastened*.” (KI9, Commissioning Stakeholder)

One of the important issues raised by the participants was licensing. The operation of vehicles either with an illegally obtained licence, or without a licence, was a serious situation which commonly involved children and underage teenagers. The lack of experience and knowledge of traffic rules in these individuals was identified as a key factor resulting in unsafe actions leading to RTCs.

*“Underage kids speeding on motorbikes…This is how we end up with traffic accidents*.*”* (KI 10, Commissioning Stakeholder)

*“Some people buy the driving licence without having any experience in doing this or even being physically able to drive*.*”* (KI 3, Service Provider)

*“People buy motorbikes for the sake of it without knowing how to drive*. *They don’t know the road signs*, *speed limits and are unable to drive*. *This is very serious*.*”* (KI 9, Commissioning Stakeholder)

The lack of knowledge of traffic rules was identified as common among other road users, irrespective of the level of experience. This in turn contributed to unsafe behaviour.

“.. *what worries me the most is that a lot of people don’t know the rules of the game*. *In other words*, *they do not know the meaning of road signals or the colours of the traffic lights*, *red or green*. *Many people don’t know this*. *They overtake on a red light*, *don’t understand minimum safe distance etc*. *All this is lack of knowledge and people need to learn this*.*”* (KI1, Service Provider)

*Interpersonal and community factors*. Participants felt that carelessness and irresponsibility was common in the community. This tended to lead to behaviour that would not only jeopardise personal safety, but the safety of others. Participants described this by illustrating the role of parents in RTCs among the young.

*“*..*underage kids with no licence*, *but worse*, *the lack of responsibility from the parents that allow them to drive*.” (KI 1, Service Provider)

*Organisational factors*. Deficiencies in policies or legislation namely in traffic control, road and vehicle engineering, were felt to be contributory to the determinants of RTCs.

“*Now there is a gas station on that side and no way to get to it without going all the way around*. *That shouldn’t be like that……*.*Those places where they sell dirty petrol and this affects the vehicles*. *There’s a lot of that with the motorbikes and it needs to be prevented*. *It shouldn’t happen that if you fill your tank half an hour later your engines stops because of the carburettor… this needs to be regulated because it’s something that’s really affecting us*… *They [the state] should be aware that this needs to be regulated because it’s something that’s really affecting us*. *And it’s not just us or Ecuador*, *this is affecting all in Latin America*.*”* (KI6, Community representative)

One participant felt that even though there were preventative strategies in the city or in the country, these were not sustained or carried out appropriately. He explained:

*“In some parts the signs on the ground are not visible anymore*. *A driver going 80 or 100 kilometers an hour and cannot see a speed bump for lack of signs or warnings*, *can end up in an accident…Police not wearing the right colours*. *In such cases the state is also to blame by not complying with the law*… *it’s not just the driver*, *but also the state by not complying with the necessary basics for prevention “*(KI 1, Service Provider)

#### Impact of RTCs

From their professional and personal experiences, participants agreed that neurotrauma is a major result of RTCs.

“*I would say [neurotrauma] is one of the most common and serious results of an accident*. *Very often we find people who suffer an accident with encephalic trauma*.” (KI3, Service Provider)

“..*the majority of patients admitted have mild to severe cranio-encephalic trauma*..*”* (KI4, Service Provider)

“*There’s a lot of neurotrauma [caused by traffic accidents]…*. *I just finished talking to a girl who had a motorbike accident…A car hit them and she did slide a long way and was out for a few minutes*, *so they brought her here*. *She now has a nervous tick and a permanent headache*.” (KI6, Community representative)

“*Lately the traumas have been very severe and many patients end up with serious neurological problems*.” (KI8, Service Provider)

“*Most accidents are motorbikes against cars and the biggest impact is on the head*.*”* (KI9, Commissioning Stakeholder)

Apart from neurotrauma, participants also identified other physical impacts of RTCs.

*“A fall from a motorbike at high speed often results in trauma to the head as well as fractures and injury to the limbs and even abdomen*.” (KI1, Service Provider)

“*Many people have died because of this problem*.*”* (KI10, Commissioning Stakeholder)

The resultant disabilities and deaths were interlinked with the non-physical impacts of RTCs.

“*Some patients remain in vegetative state*, *in coma*.*Rehabilitation is very hard and takes a lot of time putting strain not just on the clinic and the hospital*, *but on the families*.” (KI5, Service Provider)

**“***I imagine if a member of my family or myself have a traffic accident resulting in trauma*, *this has a serious impact not only on the victim but on the whole family*. *The person is left with brain damage and that is very traumatic for both the family and the injured person*.*”* (KI10, Commissioning Stakeholder)

*“There are too many accidents*, *economic losses when unable to attend work or death*. *This is all very important*.*”* (KI8, Service Provider)

### Preventing neurotrauma and RTCs in Colombia

#### Perceptions of neurotrauma prevention

Participants had an understanding of the relationship between neurotrauma and RTCs, and most felt neurotrauma prevention would be helped by prevention of RTCs, such as using protective equipment and abiding by the rules of the road. On the other hand, one participant understood prevention of neurotrauma to be providing a medical response at the time of injury.

*“I would think [neurotrauma prevention] is the prevention of trauma at the time of injury during the accident*. *For the doctor or nurse to attend the traffic accident and effectively treat the person’s injury to avoid a serious neurotrauma*.*”* (KI9, Commissioning Stakeholder)

#### Current preventative strategies and interventions

Participants had mixed responses when asked about ongoing preventative strategies and interventions for neurotrauma and RTCs in their city and country. Some felt that there were many approaches which had been developed and implemented by the traffic authorities. Others felt that there were no observable measures that were put in place to prevent neurotrauma and RTCs in their locality. There was also a general feeling that current strategies and interventions tended not to target neurotrauma specifically.

*“[Neurotrauma] interventions as such*, *there haven’t been many*.” (KI2, Service Provider)

*“I know the government has some protocols in place regarding transit security*. *But aimed at neurotrauma*, *there is nothing I am aware of*.*”* (KI5, Service Provider)

Despite this, many participants were still able to provide insight into the current strategies and interventions for RTC prevention being implemented in their respective town or city.

*Education and awareness raising*. Participants identified different ways this was being carried out. It ranged from formal education or training sessions, to more informal opportunistic approaches.

*“I’m doing some training in schools*..*We are starting a new culture with these kids and their teachers as leaders*. *We are teaching them about traffic safety as they come and leave the school*.*”* (KI1, Service Provider)

“*They now have a team of people from transport and emergency response giving talks to the adolescents about to finish high school as part of the training that is a requirement of the social work that must be done in the last years of the school*.*”* (KI6, Community representative)

“*The training [Meditech Foundation] provided for [traffic authorities and city hall representatives] last year was very good*.” (KI2, Service Provider)

“*Generally the nurses develop preventing measures*. *Things like teaching the family once the patient is admitted*, *telling them not to drink and drive*. *Basically that*, *education*.” (KI5, Service Provider)

Some participants brought up awareness raising through advertisements and campaigns.

*“*..*the national police are doing preventative campaigns at national level*. *Our controls are helping and the campaigns are on the roads and directed to people who ride motorbikes*. *They are very effective*.” (KI9, Commissioning Stakeholders)

*Legislation and enforcement*. Legislation and enforcement were felt by the participants to be important current preventative strategies. Laws relating to drink driving and overall road safety were being enforced by the traffic police through patrolling and checks on roads, particularly at busy times.

One participant also identified the role of organisations other than the police who play a role in carrying out checks on roads.

*“Well*, *when we [the Red Cross] know that there will be an increase in traffic like long weekends*, *major holidays and there is a lot of movement*, *we take to the streets*. *We try to prevent*, *take materials we give to the drivers*, *give advice and just keep monitoring the roads*.*”* (KI3, Service Provider)

*Other interventions and strategies*. Road engineering was another approach described where one participant mentioned how speed bumps were being put in place by the government. Another participant raised rehabilitation as a preventative measure carried out by those working in the healthcare sector.

#### Barriers to prevention

Participants identified existing challenges that influenced the prevention of RTCs and neurotrauma. We divided these into Individual and Community factors, and Organisational factors.

*Individual and community factors*. Participants felt that the attitude of the public made it difficult for any preventative intervention or strategy to be successfully implemented.

“..*people don’t take notice*. *People don’t take it seriously*, *they go about it all relaxed*.”(KI7, Community Representative)

“*Everybody knows the basic rules but for example*, *Colombia is one of those countries where they ignore a lot of things*. *That culture doesn’t exist out on the streets*. *Be it a bicycle or the biggest car*, *they all speed and do as they like*.*”*(KI4, Service Provider)

“*The problem is*, *people are difficult…*.*Difficult getting people to take notice of a leaflet*, *a video or audio visual with the information*, *actually telling them still doesn’t get them to show interest… the police*, *are trying to control*, *but the people won’t take responsibility*.*”* (KI9, Commissioning Stakeholder)

The participants agreed that this indifference and disinterest was most often seen among young people.

*“Young people think because they have money they can do as they please*. *It’s a matter of attitude towards the law and the consequences*. *They feel invincible and nothing will happen*.*”*(KI8, Service Provider)

The lack of knowledge and understanding was another major barrier articulated by the participants who felt that this deficiency was reflected in unawareness of preventative approaches as well as the reasons for their implementation. One participant explained:

“..[the people] *don’t actually understand these strategies*. *They think wearing a helmet is just to avoid a fine*. *They don’t see it as a safety measure in case of an accident*.” (KI8, Service Provider)

Another participant added how the lack of awareness also extended to how people responded when an RTC occurred, as illustrated by the following quote:

*“Some people never make it to the hospital because it’s not considered necessary*.*”* (KI8, Service Provider)

The lack of knowledge and awareness stemmed from the lack of education about road safety, but was also influenced by other factors such as access to such education and the baseline educational attainment of the people.

*“People don’t have access to education regarding this subject*. *There is very little about it in the media so people have no access*.” (KI5, Service Provider)

“*The level of education around here and those towns doesn’t help the people becoming aware*.” (KI9, Commissioning Stakeholder)

*Organisational factors*. Participants highlighted how governing bodies had to be held responsible for the failure in successfully preventing RTCs and neurotrauma. Deficiencies in resources, training and research, and poor planning and implementation appeared to be main barriers identified.

In terms of resources, most participants mentioned a lack of funds as an important barrier to prevention strategies. One participant also brought up the lack of manpower.

*“*..*here in […] there are very few traffic police*, *very few*. *That’s something else we need*.*”* (KI10, Commissioning Stakeholder)

One participant explained that it was not only the lack of money in general, but a lack of financial support or budgeting for prevention as a whole.

*“All the money goes into the election and then the four years in these conditions…*. *All the money they’re taking from people*, *we could use that [for prevention]*.*”* (KI6, Community representative)

Participants identified this to be the result of the indifference of the government and authorities towards prevention of neurotrauma and RTCs. Indifference and not identifying prevention as a priority resulted in a lack of support and action.

“*Prevention is in the background and nobody pays attention to it*. *Prevention would avoid accidents and in turn hospital admission*.” (KI5, Service Provider)

“*The training [Meditech Foundation] provided for us last year was very good*, *but as I said*, *traffic authority did not show up at the second seminar and the city hall had very few representatives*. *About 30 or 40 people were invited and only about 10 of us showed up*. *As you can see*, *we do not have the necessary support from the council… They don’t give priority to what’s really important*.*”* (KI2, Service Provider)

Another participant even went so far as to say that the government and authorities’ attitude also had an impact on the attitude of the public.

*“We just had a democratic broadcast…I’m saying it as I see it*, *ignorant*. *It’s my country Colombia*, *but they want to keep the people ignorant*.” (KI6, Community Representative)

A few participants felt that research and training are not considered by the government as integral components in prevention of neurotrauma and RTCs.

“*There’s not been much research on the subject [of prevention]*. *The [healthcare] professionals are not trained for that and our health structure is more focused on curing*.” (KI5, Service Provider)

Poor overall planning and implementation of strategies, manifesting in a variety of ways, were brought up by many participants: They felt that there was a lack of collective responsibility and collaboration between agencies when it came to neurotrauma and RTCs prevention.

“*I think transit [authorities] is the only entity dealing with [prevention]*. *I don’t see any politicians doing anything*. *Transit is the only one trying to do something about it*.*”* (KI7, Community representative)

*“[The Ambulance Service] don’t have [a role in prevention]*, *it’s the fire brigade in charge*.*”* (KI2, Service Provider)

The participants also raised other shortfalls in planning and implementation resulting in the relevant authorities not doing a thorough job or carrying out their responsibilities. Similarly, they felt that strategies and interventions were not being implemented uniformly throughout the country, but only in the main cities.

*“[Strategies are being implemented in] Bogota*. *I’m not sure about here in [*..*]*.*”* (KI5, Service Provider)

#### Improving prevention

Participants identified several areas where they felt improvement was needed in order to ameliorate the prevention of RTCs and neurotrauma.

*Education and awareness*. Participants felt that education should start at an organisational level where the relevant bodies should themselves be trained to educate the community, as explained by the following participant:

*“The message should be to train local bodies so that they in turn pass on their knowledge to the community*.. *I believe all authorities involved [in prevention] should jointly commit to educate the community*. *When I say community I mean everybody including pedestrians and all drivers…*.*”* (KI1, Service Provider)

When discussing the ways public education might be carried out, several participants brought up campaigns and advertisements. They felt these needed to be done more frequently, using innovative ways so that the people would take notice, remember the messages and be more aware of road safety.

“*I think we need campaigns*, *to talk about it*, *advertise*. *The more we see and hear about it*, *something will stay in people’s minds*.” (KI10, Community representative)

“*Lately*, *famous people and celebrities seem to have the most influence*. *These people could help the public become more aware*.” (KI8, Service Provider)

Many also emphasised the role of schools and how road safety education should be carried out more formally and incorporated into the curriculum.

*“I think children from a young age in school should be taught about prevention in order to avoid these types of traffic accidents*. *All schools should do it so that people are informed*, *become aware and therefore prevent these accidents*.*”* (KI10, Commissioning Stakeholder)

“*[The regional council] need to start introducing [prevention] in the initial years of school*, *even since they are children… I think there should be a class in school focusing on this subject*. *Otherwise it just gets out of hand*.. *It must be done tactically*, *with positive and realistic goals so young people don’t feel like it’s a chore*.. *They need to be monitored and made to do a course*. *The youth of today are very wild*, *like it’s their instinct*. *They need to be more sensible and have some education*.” (KI6, Community representative)

Some also suggested that such formal training measures should also be undertaken in adults, with testing after the training had been delivered.

Mobilising the right people to educate and train the public was mentioned as a productive approach to community education to ensure the message was understood, as illustrated by one participant.

“*We need to start introducing more educated people from other parts of the country telling them how to drive and control*, *but in a way that all people will understand*.*”* (KI6, Community representative)

Some participants talked about how well-timed and well-marketed educational programmes would ensure participation and subsequent success.

“*In my opinion*, *[Meditech Foundation] should do that seminar [on prevention] you once offered us but giving us more notice so that we can inform the community*, *members of the hospital and authorities*, *but with plenty of time to make people commit*.*”* [KI2, Service Provider)

“*Insist*, *insist until people turn up for those seminars [on prevention]*.” (KI10, Commissioning Stakeholders)

*Policies and enforcement*. Participants felt that more is needed to be done by the relevant parties by way of enforcement of road rules. Many felt that the traffic authorities and transit department had to be more vigilant in ensuring that laws were obeyed. Enforcement of licensing laws was thought to be an important area for the traffic authorities.

*“I think one of the main strategies should be*, *control over issuing the driving licence*. *In this country we are very lenient when it comes to this”*. (KI3, Service Provider)

*“The traffic authorities*, *the representatives of the transit department in city hall*, *should enforce all drivers both in cars and motorbikes to adhere to the basics that we mentioned*. *Firstly to have valid documentation*.” (KI1, Service Provider)

*“I think both the council and the traffic authorities need to be stricter*. *I think they should do more random stops*. *Well*, *just be out there in the streets more*. *Always ask for valid documents both to the young and the parents*. *That way they will think twice before driving without documents*..” (KI2, Service Provider)

A few participants discussed new ideas or policies that they felt should be adopted by the government and authorities to improve prevention. Suggestions included making research into RTC prevention a rule, involvement of the healthcare sector and transport companies in prevention, and health promotion and injury reduction policies. One participant also brought up the need for policies on road engineering, as explained by the following quote:

“*I was telling the secretary of transit*, *about the controls they need to put in place and improve the lanes in the busy towns where there is a lot of traffic*. *The roads need to be modified in consideration of this volume of traffic*. *They also need to open lanes to avoid many accidents*.. *There needs to be some order*, *fix and widen the roads*..” (KI6, Community representative)

*Resources and funding*. Some participants explained how the problem of resource, in particular, funds, could be mitigated. They felt it was necessary for the government to allocate a budget specific to the prevention of RTCs and neurotrauma in order to facilitate the development and implementation of necessary measures, as well as to fund research in this area.

One participant brought up another way funding could be obtained for research.

“*We could ask for financial support from the government of other countries as fortunately there are many people backing up this research*.*”* (KI8, Service Provider)

*Attitudes*, *collaboration and leadership*. Participants felt that a change in attitude of both the public and the authorities was needed to improve the prevention of neurotrauma and RTCs. It was felt that both parties have to assume more responsibility and care more about prevention. They agreed that there is a need for everyone to realise the seriousness of RTCs and neurotrauma, and to be committed to prevention in order to ensure all the relevant strategies and interventions were not only implemented nationally, but were successful.

It was also felt that solid relationships and collaboration within the relevant entities such as the transit authorities, educational bodies and health workforce, are also necessary in the planning and implementation of successful preventative strategies and interventions.

At the same time, the participants felt that the relevant authorities should also engage with the community to allow for a better understanding of the problem and work together to solve it. One participant brought up the concept of ‘citizens participation’ where members of the public can be involved in development efforts relating to prevention.

Some participants described how new leadership could play a role in improving the prevention of neurotrauma and RTCs. One participant said:

*“Well*, *we are about to have a new Mayor*. *We hope that it will be someone who can feel our town’s pain and wants the town to grow*. *Someone who regardless of politics*, *will care and educate the youth of this town… What we need is somebody with ideas and to implement them*.*”* (KI2, Service Provider)

#### Role of research

*Awareness of current research*. Differing opinions arose when participants were asked about any current research in neurotrauma and RTCs prevention in their city and country. Some were unaware of any research being done, whereas others felt there was none being done at all. Two participants identified the Meditech Foundation as the body that is undertaking research in this area.

*Research priorities*. Most participants agreed that research is an essential component of prevention, and described several areas they felt was important. Only one participant did not know what kind of research would be necessary to reduce RTCs and neurotrauma.

The priority areas are given below:

Causes of RTCs

Several participants felt it was necessary to find out the causes of RTCs.

*[“Research] is very important*. *To find out why the accidents continue*, *why so many dead and injured is very important*.*”* (KI1, Service Provider)

Consequences and impact of RTCs

Research into the various consequences of accidents was as important as investigating the causes, as explained by one participant:

Interviewer: *What kind of research should be done in order to prevent traffic accidents leading to neurotrauma here in [*..*] and in the whole country*?

Participant: *The social and economic impact resulting from deaths and neurological disabilities can be a focal point for the city and the whole country*…*Maybe use a real case from a patient…[Find out] previous deaths*. *Deaths before arriving to hospital and deaths once in hospital receiving attention*. *Surviving up to five years and quality of life of a clinical patient*.. (KI8, Service Provider)

Involving the public in research

The participants felt that research should involve members of the public, and should determine their understanding and perceptions of RTCs, and the various issues surrounding RTCs.

“*I think the research should focus on the people*. *Is important you ask the public in order to find out what they know and in turn learn what to add to the research…Find out the public’s perception about what a traffic accident is*. *If the people know the Highway Code*. *If the people are aware of the consequences of an accident*. *I think that’s needed*.*”* (KI5, Service Provider)

Improving prevention

Some participants identified the role of research in improving prevention by looking into best practice as well as evaluating pre-existing strategies to determine whether they worked or not.

“*I think [education] should be researched so as to do it properly*.”(KI5, Service Provider).

“*Research is very important*. *It will improve the education in the community*, *families and schools so they understand the problem… There needs to be a plan and make it work… Look at which strategy worked or not*. *Do we do this or the other*, *why did that happen*, *etc*. *“*(KI6, Community Representative).

*Dissemination of research findings*. Participants agreed that research could improve prevention when public knowledge and awareness is increased through the sharing of research findings with end-users. They identified several ways this could be done: Most felt that the mass media, including social media, was the best way for such information to be shared due to the extent of usage and coverage.

Some felt that specific entities, namely educational bodies, healthcare providers, policy-makers and law enforcement should develop other means of disseminating research, as illustrated by the following quotes:

*“[The local council] host many groups like sports*, *educational groups*, *work groups and within these we could program something*. *Once we see the people*, *assess their education level or interests*, *then we could introduce this*. *Sports are big here*, *volleyball*, *they have won many matches*, *so that’s a way to introduce [research results]*.*”* (KI6, Community representative)

Interviewer: *How do you think we could best share the results of the [research] in […] and the country in general*?

Participant: *Get everybody together*, *link the entities like a chain*. *Hospitals*, *City Hall*, *Commissioner*, *transit police*, *all schools… everybody should have a manual explaining why they should wear a helmet*, *why they should follow the rules*. *I think that can be a way of preventing and reducing the many accidents both at county and national level*. (KI10, Commissioning Stakeholders)

Another participant identified yet another way information could be disseminated.

“*I think for those who have motorbikes*, *they should receive a call and inform them of the [research] results*. *Or even at the traffic lights*… *I think the phone calls would be good as those people are so unaware*.*”* (KI7, Community Representative)

## Discussion

This study sought to explore local contexts and perspectives on RTCs and neurotrauma, utilising the views of key informants from different localities in Colombia. As far as we know, this is the first study to do so in Colombia, although similar studies have been carried out in other LMICs, looking into various aspects of road safety [31–33].

From the interviews, it is clear that RTCs and neurotrauma from RTCs are perceived to be a major problem across Colombia, and motorcycles are believed to be most frequently involved. These perceptions are supported by a recent World Health Organisation report which showed that, of the 13.4 million vehicles in Colombia, more than 50 percent (7.5 million) are motorcycles [29]. These findings are also consistent with the current literature on typical victim profiles in LMICs, where RTCs are more common in two or three-wheeler riders and pedestrians [34, 35].

The relationship between RTCs and neurotrauma was clearly illustrated by the participants when they acknowledged that neurotrauma was one of the most common injuries from RTCs, despite the absence of documentation on head trauma and RTCs in Colombia [20, 36]. The findings also demonstrate links between the determinants and impact of RTCs, when participants were able to identify the social and economic implications of death and disability from RTCs in the context of neurotrauma being the most common injury sustained by victims. In Colombia, as with other LMICs, one of the groups with the highest deaths and injuries from RTCs are adults under the age of 40 [20, 23, 37, 38]. As most who are affected are men, and given the gender disparities in economic opportunities, the financial loss to families directly, and to nations, indirectly, is great, [37, 39, 40]. Some participants also highlighted the strain to healthcare systems. In Colombia, the healthcare expenditure for injuries from RTCs is estimated to be between 1.6% and 4.2% of the GDP, with annual costs exceeding USD 1 billion [37, 40]. In addition, the burden often exceeds the manpower and facilities available to manage the patient load [23, 37].

When discussing the causes of RTCs, participants identified contributing human and organisational factors in their city and country. These factors include unsafe actions of speeding and drink driving occurring in conjunction with unregulated, improper licensing practices. The latter appears to be the result of indifference by parents allowing their underage children to operate motor vehicles without a licence, and indifference by the relevant authorities to enforcing licensing laws. These views suggest that there is a lack of skill, inexperience and risk-taking behaviour, the ‘dangerous triad’, that results in RTCs, especially in the young [41].

Participants also discussed personal protective equipment in relation to RTCs. Given their understanding of the close link between RTCs and neurotrauma, as well as the predominant vehicle on the road being the motorcycle, participants described helmet use in the population, which was found to be lacking. This is not new where recent studies conducted in Colombia have indicated that despite laws on the mandatory use of motorcycle helmets, there have been problems surrounding enforcement and overall compliance with this legislation [37, 42]. Qualitative work with motorcyclists in certain cities in Colombia has also highlighted how there is the perception that helmet use is for avoidance of penalties, rather than for safety- another issue that was evident from our findings [42]. The improper use of helmets that was brought up in the interviews could be due to the fact that helmet laws in Colombia do not meet best practice, where helmet fastening is not required by law [35].

Carelessness and irresponsibility were identified as the major underlying behaviours that seemed to result in an RTC-prone society. This was believed to arise from a negative attitude of the people towards road safety, which was not taken seriously and largely ignored. This attitude was also perceived to occur in the government and relevant authorities, which participants felt influenced and perpetuated the public’s attitude. Additionally, the indifference seen in the authorities was evident in the lack of action when it came to prevention of RTCs, where resources were not appropriately allocated, and research and training was inadequate, resulting in deficient or irregularly implemented interventions. It was also interesting that participants felt that there were no clear strategies in place to prevent neurotrauma. These findings complement those in the current evidence-base which indicate that LMICs tend to lack robust preventative programmes particularly in secondary and tertiary neurotrauma prevention, often due to paucity of resources and research, which were also identified in our study as barriers to prevention [6, 7, 11, 12, 43]. However, setting aside a budget for prevention and seeking external funding were suggested as ways to address these issues, where the latter would be carried out in the context of research [9].

The lack of organisational collaboration and coordination has long been recognised as a barrier to prevention of RTCs, and thus neurotrauma [23, 44, 45]. Despite there being a National Agency for Road Safety that was set up in Colombia in 2015, participants felt that there should be more entities taking responsibility for prevention and that there should be an interactive, collaborative planning and management of road safety which also involves the community [23, 39].

Involvement of both the government and civil society in road safety has been strongly encouraged by the World Health Organisation (WHO) and has contributed significantly to positive outcomes in various road safety initiatives worldwide [46]. This appears to be emerging in Colombia, based the findings of this study, where there have community initiatives to educate school students and the concept of citizens being involved in planning and development. In addition, local reports have indicated that in Bogota, the mayor has introduced policies where the public may also contribute to reforms to reduce the road fatality rate, for example, by reporting non-adherence to traffic rules [47].

Despite conflicting results in the literature, and debates about what constitutes ‘education’ (whether it is knowledge acquisition or skill acquisition), educational interventions play an important role in the prevention of RTCs and neurotrauma [32, 48]. This was demonstrated in our results where participants described how education was being carried out in the country, but also brought up the need for regular, consistent multimodal programmes that are engaging, well-organised and targeted. Given the disparities in educational attainment in Colombia, such strategies should be tailored to the target audience, and carried out by experienced and qualified people.

The participants emphasised how education, of both the public and relevant authorities, would serve as a source of knowledge leading to awareness and improved skills, and even result in a change in attitude and behaviour, especially in young people. Indeed, approaches that seek to educate and increase awareness are based on changing attitudes, which would lead to behavioural change [39]. It is this nationwide change in attitude and behaviour that participants felt was a necessary ingredient for the improvement of any preventative efforts in the country.

The concern for the young was further illustrated by the suggestion to introduce road safety education in schools as part of the curriculum. As with many LMICs, Colombia does not have any formal road safety education in schools [39]. This educational approach is not new and is based on the understanding that road safety education tends to be more effective when started at a young age [49, 50]. Continuing this in secondary school ensures that education is sustained and addresses different age-appropriate aspects of road safety [50]. Although there has been criticism as to the effectiveness of this strategy, where outcomes are measured in terms of increased knowledge and skill rather than event reduction, road safety education enables the young find out about the road environment and how it functions, how they can influence changes in that environment, and most importantly, how to keep themselves and others safe both now and in the future [48, 51, 52]. Additionally, education carried out in conjunction with the other ‘E’s’, namely enforcement and engineering has been shown to be most effective in reducing RTCs [32, 50]. From the results, these other strategies appear to be implemented in Colombia, but participants felt more action had to be taken by the authorities to ensure these approaches brought about the desired outcome.

The involvement of celebrities and famous people in various health and social causes has been on-going for years [53, 54]. Despite controversies, particularly pertaining to evidence on effectiveness, many countries have utilised this method to raise awareness, especially amongst young people [54, 55]. In Colombia, celebrities have led or been ambassadors for various campaigns and although there have been none for road safety, it appears that this is something that participants’ felt was worth exploring as their local celebrities seemed to be influential enough to garner public interest and awareness [56]. This would also allow for both community and media involvement in prevention, which were identified to be lacking by the participants. Furthermore, efforts like this would also involve government or political entities directly, through organisation of such campaigns, or indirectly, when such campaigns would influence leaders to take action. This now would be an example of collaboration between agencies and civil society, where collective responsibility for road safety would enable prevention to be prioritised in the country.

The areas identified by participants as research priorities were consistent with those recommended by researchers and experts in the field of road safety and neurotrauma [6, 11]. In keeping with the theme of community involvement in prevention, participants also discussed public involvement in research, an important component that is still fairly new in LMICs [57, 58]. Looking into all these research areas would be helpful for targeting resources and developing evidence-based but context-appropriate interventions that would be also assessed for effectiveness [13, 23, 59, 60].

### Strengths and limitations

To our knowledge, this is the first study utilising qualitative methods to gather information on the local circumstances relating to RTCs and neurotrauma in Colombia, and to identify ways of improving the situation. This study is an example of participatory or action research, where there is collaborative co-creation of knowledge between the researcher and knowledge users [61, 62]. This approach facilitates the development of solutions which are not only evidence-based, but impactful and sustainable, which is particularly important in resource limited contexts [13, 62, 63].

Despite carrying out peer checking to reduce bias in analysis, member checking could not be done as the transcripts and results are in English. As most of the participants are not familiar with the language, it would have been difficult to determine if our interpretation truly represented what they wanted to say, especially since translation was carried out by a translator and not the researcher. However, this issue was somewhat addressed as transcripts were checked for accuracy by the interviewer who is fluent in both languages.

Another limitation was the small sample size (n = 10). The fact that this study was just one part of a larger project that needed completion in order for the next part to commence, affected the recruitment process due to time limitations. Despite efforts from the local team to garner interest, there appeared to be a general lack of enthusiasm in participating in research amongst potential participants. Issues pertaining to the research agreement between the Cambridge and Colombian teams also made it difficult to seek help from other relevant organisations for recruitment. Although this study is purely exploratory, and there was no intent on involving a large number of participants, it is still possible that the sample size was limited, and could potentially affect the interpretation of our findings.

Nevertheless, we were able to carry out Interviews with a broad range of key informants involved in various aspects of prevention, allowing diverse perspectives on the topic. However, the participants came from select towns and cities in the country, where there was no representation from other localities, including the capital city, Bogotá. Although the participants were fairly knowledgeable about the situation in Colombia, there was a lot of reference to issues in their own city or town, which may not be generalisable to other parts of the country. Hence, the results should be considered with some caution.

### Recommendations

Based on the findings of this study, several practical strategies can be recommended to improve prevention of RTCs and RTC-related neurotrauma in Colombia. These strategies, which generally target governments and policy-makers, are summarised in Table 3.

**Table 3:** Table of recommended strategies for improving prevention of RTCs and RTC-related neurotrauma in Colombia.

Road safety management and leadership	• Ensuring a yearly budget allocation for prevention
	• Seeking external support or funding where necessary, especially for research
	• Sounder policies on road safety and injury reduction, including road and vehicle engineering, to be implemented throughout the country
	• Ensuring beneficial policies and legislation are sustained and implemented consistently
Education and awareness-raising	• Regular training on prevention for relevant government entities, including healthcare professionals
	• Emphasising prevention in education programmes for healthcare disciplines
	• More frequent campaigns and advertisements, utilising the media and members of the community
	• Introducing road safety education as part of the school curriculum for both primary and secondary schools
	• Establishing regular, frequent and well-publicised education programmes tailored to the community, led by qualified individuals
Enforcement of laws	• Stricter enforcement of traffic laws, particularly in the area of licensing
Collaborations and Partnerships	• Forging and strengthening inter-departmental and inter-organisational collaborations, especially with transport companies
	• Involving the public in planning and implementation of preventative strategies as well as in research
Research	• Encouraging and supporting research around RTCs, neurotrauma and prevention
	• Ensuring research findings are disseminated to the public through different media outlets or specific programmes

## Conclusion

This study explored the perspectives of key informants on RTCs and neurotrauma in Colombia. Although participant views focussed primarily on RTCs, they were clear that it is a major cause of neurotrauma. This study illustrates that RTCs still remain a big problem in the country despite the current preventative strategies and interventions. It also draws attention to the human and organisational factors that contribute to the problem and pose challenges to prevention, as well as possible ways these could be rectified. These findings have the potential to influence policy and practice by providing solutions from community members who are knowledgeable in this area. This study also highlights the need for further research to attain more objective measures of the problem and the effectiveness of current preventative strategies.

## Supporting information

S1 File (DOCX)
